# Light scattering in human dentin

**DOI:** 10.1364/BOE.590212

**Published:** 2026-06-01

**Authors:** Levin Stolz, Alwin Kienle, Sascha Hein, Katrin Heck, Elias Walter, Florian Foschum

**Affiliations:** 1 Institute for Laser Technologies in Medicine and Metrology, Helmholtzstr. 12, 89081 Ulm, Germany; 2 Ulm University, 89081 Ulm, Germany; 3 Emulation S.Hein, Rennweg 17, 79106 Freiburg im Breisgau, Germany; 4 Department of Conservative Dentistry, Periodontology and Digital Dentistry, University Hospital, LMU, Munich, Germany

## Abstract

Understanding light propagation in human dentin is critical for dental diagnostics, including optical coherence tomography (OCT) and early caries detection. Dentin’s heterogeneous microstructure—a dense network of cylindrical tubules ( ≈2μm diameter) embedded in an organic–inorganic matrix—induces both isotropic and anisotropic light propagation. Previous isotropic-only models cannot capture the pronounced angular dependence observed experimentally, leading to substantial errors in the estimation of optical parameters. We present a combined isotropic–anisotropic Monte Carlo model for light transport in dentin, incorporating directionally dependent propagation in tubules based on Maxwell’s equations for infinitely extended cylinders. Optical parameters were determined from angular-resolved goniometric measurements on three human teeth across wavelengths from 400 nm to 700 nm, with controlled tubule orientations. Using particle swarm optimization to fit the model to experimental data, we quantitatively characterized both propagation regimes: the intertubular matrix ( $\mu_{a}\approx 0.5\;{\text{mm}}^{-1}$ , $\mu_{s}\approx 1.8\;{\text{mm}}^{-1}$ , *g* ≈ 0.85) and tubule geometry (diameter 2.25 µm, areal density 19 000 mm^−2^ to 60 000 mm^−2^). Comparison with an isotropic-only model revealed that neglecting anisotropic propagation introduces absorption errors exceeding 40 %, emphasizing the importance of the combined approach. This framework enables robust inverse problem solving for dental optics and can be extended to study other tissues exhibiting anisotropic light propagation, such as brain white matter, tendon, and muscle.

## 1. Introduction

A detailed understanding of light propagation in tooth tissue is essential for many dental applications, including advanced diagnostics such as optical coherence tomography (OCT) and caries detection, therapeutic laser treatments, and the optical matching of restorative materials to natural teeth. Among dental hard tissues, dentin presents a particular challenge regarding the comprehension of light propagation due to its heterogeneous structure, which introduces both isotropic and anisotropic effects on light transport [1,2].

Dentin consists of a dense network of microscopic cylindrical tubules, approximately 1 µm to 3 µm in diameter, which radiate roughly radially from the dental pulp toward the exterior of the tooth [3–7]. These tubules are embedded within a composite organic-inorganic matrix (intertubular matrix), composed largely of collagen and hydroxyapatite. Owing to their high density and regular alignment–estimated at around 15000 to 65000 tubules per mm 2 depending on location [3,5]–the dentin tubules act as efficient optical scatterers. The spatial orientation of the tubules gives rise to strongly direction-dependent anisotropic light propagation [8–10], whereas the intertubular dentin provides a more homogeneous, isotropic light diffusion.

A number of studies have shown that neglecting this anisotropic light propagation leads to incomplete or inaccurate descriptions of dentin’s optical behavior. Purely isotropic scattering models are unable to reproduce the strong angular dependence observed in transmission and backscattering measurements [10]. This has practical implications: in OCT, anisotropy affects image contrast and fidelity, particularly for features oriented obliquely to the illumination [11,12]. In caries diagnostics, early demineralization alters scattering properties in ways that can be obscured by tubule-induced anisotropy [13,14]. Moreover, achieving realistic optical appearance in restorative materials requires not only spectral color matching, but matching of scattering anisotropy as well [15].

Previous work has addressed individual aspects of this problem. Early models treated dentin as an isotropic medium [16,17], while later studies demonstrated that the primary scattering contribution originates from the tubules rather than from inhomogeneities within the intertubular matrix [10,18]. Subsequent theoretical and experimental investigations modeled the tubules as infinitely long cylinders, enabling quantitative descriptions of anisotropic scattering using solutions of Maxwell’s equations [8,9,19]. In parallel, bulk optical parameters have been determined from collimated beam extinction measurements [1,20].

Following the line of research by Kienle et al. [21], the present work introduces a combined isotropic-anisotropic model for light transport in dentin. We embed the directionally dependent scattering predicted by Maxwell’s equations for optically infinitely extended cylinders within an isotropically scattering matrix. In contrast to previous approaches, we determine the optical properties of both scattering regimes experimentally using goniometric measurements. This integrated model more accurately captures the physiological structure of human teeth and provides a comprehensive description of light transport in dentin.

## 2. Materials and methods

### 2.1. Specimen

Three human tooth slices previously prepared and described by Pink et al. [22] were used in this study. The specimens originated from teeth extracted for orthodontic or periodontal reasons, following informed consent procedures as detailed in the original publication.

Sample preparation followed the protocol of Pink et al. [22]. After cleaning and polishing with pumice, the teeth were stored in a 0.1 % thymol solution until processing. Vertical labio-/bucco-lingual slices were obtained using a water-cooled low-speed diamond saw (IsoMet LS, Buehler, Lake Bluff, IL, USA). The slices were then ground and polished to achieve thin, optically smooth sections suitable for optical measurements. Fine grinding was performed with 1200 and 2500 grit silicon carbide papers, followed by multiple polishing cycles using diamond suspensions of 3 µm, 1 µm, 0.25 µm, and 0.05 µm particle size. After polishing, all samples were cleaned ultrasonically in distilled water.

The three specimens used in the present study were:

• **Tooth A:** premolar, donor age 13.1 years, thickness 0.238 mm
• **Tooth B:** premolar, donor age 10.4 years, thickness 0.136 mm
• **Tooth C:** incisor, donor age 65 years, thickness 0.14 mm

All samples were handled and stored under identical conditions to preserve surface quality and hydration state.

### 2.2. Goniometric measurement of light scattering

Light scattering was characterized using an optical goniometer. The goniometric measurements were performed with the optical setup described in [23,24], which enables precise detection of scattered light from a quasi-monochromatic source (Mountain Photonics Hyperchromator II) within the incident plane over a full angular range of 0° to 360°. The incident beam was focused onto the sample surface at normal incidence, yielding a spot size of 1.35 mm. Scattered and ballistic light were recorded by a rotatable CMOS camera (IDS U3-3060CP-M-GL Rev2.2), positioned such that its entrance pupil of 5 mm was 120.8 mm from the rotation center. The optical system was optimized to suppress stray light and ensure high-precision angular detection.

**Fig. 1.**
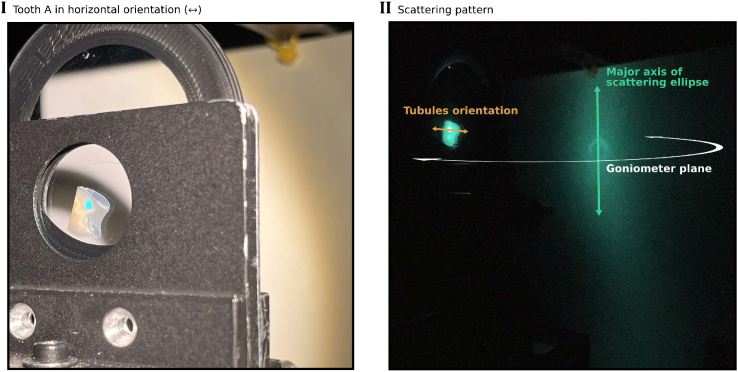
Photographs of the goniometric measurement setup. **I** Tooth A in horizontal orientation inside the glass cuvette. **II** With ambient illumination turned off, the approximately vertical scattering pattern of the dentinal tubules, having a mostly horizontal orientation, can be observed.

For clarity, the orientation parallel to the goniometer plane (see Fig. 1) is defined as horizontal ( ↔ ), and the perpendicular orientation as vertical ( ↕ ), as illustrated in Fig. 1. Each specimen was measured twice per wavelength—once with the tubules approximately aligned horizontally and once vertically. Alignment was achieved by illuminating the sample at perpendicular incidence relative to surface of the dentin slab and observing the transmitted and scattered light pattern on a flat screen placed behind the sample. The dentinal tubules produced an elongated, elliptical scattering pattern; to a first approximation, the tubules in the considered slab are oriented perpendicular to the major axis of this ellipse. The specimen was rotated until the major axis was aligned horizontally or vertically, corresponding to tubule orientations of vertical ( ↕ ) and horizontal ( ↔ ), respectively.

Each tooth specimen was placed in a glass cuvette filled with distilled water to preserve hydration and minimize the influence of the remaining surface roughness on the goniometric measurements. The cuvette was assembled from two nBK7 glass slides separated by a spacer ring slightly thicker than the tooth slice.

Measurements were performed at wavelengths of 400 nm, 500 nm, 600 nm and 700 nm. The spot of the incident beam was positioned approximately midway between the pulp and enamel, primarily probing mid-coronal dentin. All measurements and simulations, see next section, were normalized to the total incident light for each wavelength. This was achieved by performing a reference measurement and simulation with an empty sample stage and the detector set at direct transmission.

### 2.3. Computational modeling using Monte Carlo simulations

Light propagation within the tooth structure was modeled using a Monte Carlo (MC) simulation framework of the radiative transfer equation incorporating a detailed digital optical twin of the experimental setup. The model accounted for all relevant optical components, including optical fibers, lenses, apertures, the sample holder, and the cuvette geometry composed of nBK7 glass panels and water layers between the cuvette and specimen. A comprehensive description of the digital optical twin is provided in [23,24]. The framework is implemented in C+ + and OpenCL and was executed on eight parallel NVIDIA A10 GPUs.

The tooth specimen was represented by a nested cylindrical geometry, with an inner cylinder corresponding to dentin and an outer shell representing enamel. The enamel layer was modeled as an isotropic medium with optical parameters $\mu_{a}=0.1\;{\text{mm}}^{-1}$ , $\mu_{s}=15\;{\text{mm}}^{-1}$ , g=0.94 , and n=1.62 [17,22,25,26].

Dentin, in contrast, exhibits pronounced anisotropic light propagation due to its microstructure. As mentioned above, it consists of a dense alignment of microscopic channels, known as dentinal tubules, which extend, in first order, radially from the pulp chamber towards the enamel-dentin junction. This highly organized structure significantly influences light transport, as the tubules act as partially guiding scatterers that preferentially scatter photons along a cone around their longitudinal axes.

The intertubular matrix was modeled with isotropic light propagation, which is characterized by an absorption coefficient $\mu_{a}$ , a scattering coefficient $\mu_{s,\text{isotrop}}$ , a refractive index $n_{m}=1.52$ , and the Henyey-Greenstein phase function [27] with anisotropy parameter g , all assumed to be independent of the incident angle. The dentinal tubule radii R were modeled as log-normally distributed via the dimensionless variable

(1) $$x_{r}:=\ln\left(\frac{R}{R_{0}}\right)∼N(\mu_{r},\sigma_{r}^{2}),$$

with $R_{0}=1\;\mu \text{m}$ . The model parameters are $\mu_{r}$ and $\sigma_{r}$ , together with the tubule volume fill factor ϕ , a refractive index of $n_{t}=1.33$ , the mean orientation vector $\vec{o}$ , and an angular deviation of the orientation described by a two-dimensional Gaussian distribution with standard deviations $\sigma_{1}$ and $\sigma_{2}$ . $\sigma_{1}$ describes a tilt out of the plane of the tooth slice while $\sigma_{2}$ describes a tilt in the plane of the tooth slice.

Due to their elongated, cylindrical shape, the scattering coefficient and phase function of the tubules depended on the photon’s incident angle relative to the tubule axis. Angle-dependent scattering parameters were derived from the analytical solution of Maxwell’s equations for the scattering of a plane wave by an infinitely long dielectric cylinder [28,29]. The log-normal radius distribution was discretized into 11 sampling points spanning the 95 % confidence interval. For each radius, scattering coefficients (accounting for the tubule fill factor) and phase functions were precomputed and stored in a lookup table (LUT) covering incident angles from 0° to 90° in 0.5° increments.

Photon propagation in the isotropic regions followed the standard MC approach. For the dentin layer, showing anisotropic light propagation, the calculation was handled as follows: A photon traveling within the dentin with propagation direction $\vec{v}$ encountered tubules with orientation vector $\vec{o}$ . The vector $\vec{o}$ was randomly perturbed according to the Gaussian angular distributions given by $\sigma_{1}$ and $\sigma_{2}$ . The incident angle between $\vec{v}$ and the perturbed orientation $\vec{o}$ was computed, and the corresponding anisotropic scattering coefficient $\mu_{s,\text{anisotrop}}$ was obtained by interpolation from the LUT. The photon path length p was then sampled as

(2) $$p=-\frac{\ln(\zeta )}{\mu_{t}},\;\text{where}\;\mu_{t}=\mu_{a}+\mu_{s,\text{isotrop}}+\mu_{s,\text{anisotrop}},$$

with ζ denoting a uniformly distributed random number in (0,1) .

If scattering occurred, the type of scattering event was determined probabilistically. Scattering by the anisotropic component was selected with probability

(3) $$P_{\text{anisotrop}}=\frac{\mu_{s,\text{anisotrop}}}{\mu_{s,\text{isotrop}}+\mu_{s,\text{anisotrop}}},$$

in which case the phase function and deflection angles were obtained by interpolation from the LUT and the photon’s new direction and position were updated accordingly. Otherwise, isotropic light propagation was assumed applying the coefficient $\mu_{s,\text{isotrop}}$ and the Henyey-Greenstein phase function with parameter g .

Each forward simulation tracked approximately $3\times 10^{7}$ photons, which was found to give a stable goniometric signal over the full 360° angular range down to signal levels of ${\approx 10}^{-6}$ of the incident intensity. A single forward simulation took approximately 7 s on the GPU cluster.

### 2.4. Particle swarm optimization

To determine the optical parameters that best reproduce the measured scattering data, a particle swarm optimization (PSO) algorithm was employed. The optimization used 60 particles over 50 iterations, with each particle’s local neighborhood limited to the 12 nearest particles (Euclidean distance). The inertia weight was set to w=1.2 , with cognitive and social acceleration coefficients $c_{1}=c_{2}=1.5$ . In each iteration step, two forward simulations were performed per particle, corresponding to the horizontal and vertical tubule orientations. The measured data from both orientations were fitted simultaneously within each optimization step. This corresponds to 60×50×2=6000 forward simulations per wavelength and specimen, giving a total runtime per wavelength of approximately 12h on the GPU cluster.

The cost function was defined as the sum of squared differences between measured and simulated goniometric curves on a logarithmic intensity scale, weighted equally across both tubule orientations and the full angular range.

The set of fitted parameters included $\mu_{a}$ , $\mu_{s,\text{isotrop}}$ , and g , representing absorption, scattering, and phase function parameter for the isotropic light propagation within the dentin matrix. The contribution to the light propagation due to the dentinal tubules was fitted with the median tubule radius $r=R_{0}\exp(\mu_{r})$ (the median of $x_{r}$ ), the tubule volume fill factor ϕ , the z -component of the tubule orientation vector ${\vec{o}}_{z}$ , and the angular perturbation parameters $\sigma_{1}$ and $\sigma_{2}$ . The standard deviation of the tubule radius was fixed at $\sigma_{r}=0.1\exp\mu_{r}$ . The lateral orientation components were assigned according to the measurement configuration: ${\vec{o}}_{x}=1$ and ${\vec{o}}_{y}=0$ for the horizontal orientation ( ↔ ), and ${\vec{o}}_{x}=0$ and ${\vec{o}}_{y}=1$ for the vertical orientation ( ↕ ).

The search bounds were chosen to comfortably bracket the physiological range of dentin parameters: $\mu_{a}\in [0.01,3]\;{\text{mm}}^{-1}$ , $\mu_{s,\text{isotrop}}\in [0.05,10]\;{\text{mm}}^{-1}$ , g∈[0.5,0.9999] , tubule radius r∈[0.5,3]μm , ϕ∈[0.05,0.55] , $\arctan({\vec{o}}_{z})\in [1^{\circ },40^{\circ }]$ and $\sigma_{1},\sigma_{2}\in [0.1^{\circ },15^{\circ }]$ .

Each wavelength was fitted independently. Since the parameters characterizing the tubule structure (anisotropic contribution) are expected to remain constant with wavelength, mean values as well as maximum and minimum deviations were computed from the fitted results.

To verify the uniqueness and stability of the global minimum, all particle positions with a cost function value less than 25 % above the global best were analyzed. For all specimens and wavelengths, the maximum deviation of the fitted parameters from the global best solution was below 8 %, typically less than 1 %. These results indicate the absence of secondary minima yielding equivalent fits to the data.

## 3. Results

### 3.1. Characterization of combined isotropic and anisotropic light propagation

**Fig. 2.**
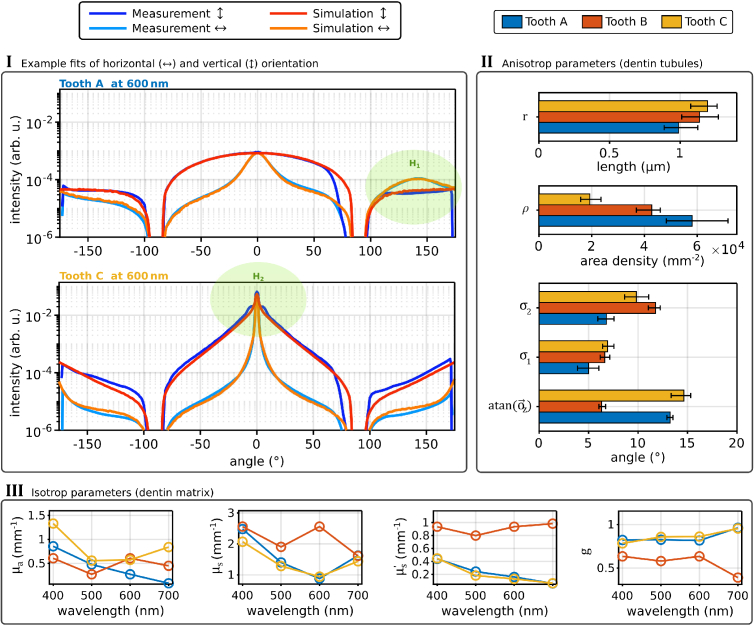
Characterization of combined isotropic-anisotropic light propagation model. **I** Two representative best-fit examples of the goniometric signal. **II** Geometric parameters of the dentin tubules, capturing the anisotropic scattering contribution. **III** Fitted optical parameters of the intertubular dentin matrix, representing isotropic light propagation.

Figure 2 summarizes the fitting results for all three specimens. The areal density ρ of dentinal tubules was calculated from the fitted parameters ϕ and ${\tilde{\mu }}_{r}$ according to

(4) $$\rho =\frac{\phi }{\sum_{i=1}^{11}P(r_{i})\pi r_{i}^{2}},$$

where $r_{i}$ represents the 11 discretized tubule radii, and $P(r_{i})$ is the probability derived from the fitted log-normal distribution.

Panel I of Fig. 2 presents two representative best-fit examples of measurements on two tooth slabs. Transmission through the specimen is observed between −90° and 90°, where the ballistic (unscattered) transmission peak appears at 0°. Reflection is detected in the angular ranges of 90° to 180° and −90° to −180°. When the tubules are oriented vertically, increased scattered light intensity is detected, as light scattering from cylindrical structures confines scattering within the cone defined by the incident direction rotated about the cylinder axis. In the upper fit, a region of enhanced scattering for horizontal tubules is highlighted with green color ( $H_{1}$ ). This effect can be achieved by forward-tilting the tubules (increasing ${\vec{o}}_{z}$ ). This causes horizontally aligned cylinders to scatter light toward only one side of the reflection hemisphere, similar as a mirror. The lower fit shows interference maxima near the transmission peak (H 2 ), further detailed below.

Panel II of Fig. 2 shows the fitted parameters characterizing the tubules, averaged across all wavelengths. Error bars indicate the minimum and maximum values over the spectral range. Measurements are predominantly influenced by the mid-dentin region between pulp and enamel. The mean tubule diameter across specimens is 2.25 µm, with minor variation. The area density ranges from approximately 19 000 mm^−2^ to 60 000 mm^−2^, with the lowest density corresponding to incisor tooth C.

**Fig. 3.**
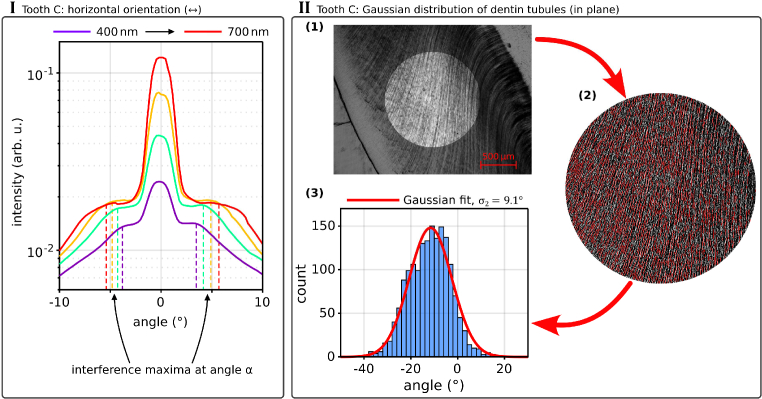
Details on the dentin tubules parameters. **I** Wavelength-resolved signal near the transmission peak for the horizontal orientation of tooth C, showing distinct interference maxima. **II** Estimation of the in-plane angular distribution of tubules within the illuminated region of the microscope image. Tubule orientations are identified from the image (1), fitted with straight lines (2), and the resulting angle distribution is modeled with a Gaussian (3).

As indicated by H 2 in Fig. 2 I and shown in detail in Fig. 3 I , vertical orientation measurements of tooth C exhibit interference maxima at angle α . Assuming these are first-order diffraction maxima from the vertically aligned tubules, the corresponding lattice constant d is given by

(5) $$d=\frac{\lambda }{\sin(\alpha )},$$

which averages to d=6.9μm across all wavelengths. This corresponds to an area density of

(6) $$\rho_{\text{interference}}=\frac{1}{d^{2}}\approx 21000\;{\text{mm}}^{-2},$$

which is in good agreement with the fitted density of $\rho =19289\;{\text{mm}}^{-2}$ for tooth C. The fitted values of $\tilde{\mu_{r}}$ and ρ are also consistent with previous SEM studies [3,7].

The parameters $\sigma_{1}$ and $\sigma_{2}$ represent perturbations of the tubule orientation, modeled as normal distributions. Given the 1.35 mm diameter of the illuminated area, measured values reflect an average over local structural inhomogeneities. Figure 3 II shows this in detail: step (1) depicts a transmitted light microscope image with the approximate illumination spot; step (2) identifies directional features within this spot and fits line segments to the tubules; step (3) fits the angular distribution of tubules relative to the vertical axis with a normal distribution, yielding $\sigma_{2,\text{microscope}}=9.1^{\circ }$ . This agrees well with the fitted in-plane perturbation of $\sigma_{2}=9.8^{\circ }$ for tooth C.

Panel III of Fig. 2 presents the fitted optical parameters of the intertubular dentin matrix, modeled as an isotropic medium. The absorption coefficient ranges from 0.2 mm^−1^ to 1.4 mm^−1^, with higher absorption observed at shorter wavelengths. The scattering coefficient of the intertubular matrix varies between 1 mm^−1^ and 2.8 mm^−1^ across the examined wavelength range. The anisotropy parameter g of the Henyey-Greenstein phase function was determined to be approximately 0.89 for teeth A and C, whereas tooth B exhibited a notably lower value of g≈0.6 .

To quantify the relative scattering contributions from the dentinal tubules and the intertubular matrix, we derived the average anisotropic scattering coefficient ${\bar{\mu }}_{s,\text{anisotrop}}$ for each specimen. This parameter was determined by tracking all scattering events of all photons attributed to the tubules during the photon transport simulations. For the three specimen we obtained

(7) $${\bar{\mu }}_{s,\text{anisotrop, tooth A}}\approx 250\;{\text{mm}}^{-1},$$

(8) $${\bar{\mu }}_{s,\text{anisotrop, tooth B}}\approx 180\;{\text{mm}}^{-1}$$

(9) $$\text{and}\;\;\;{\bar{\mu }}_{s,\text{anisotrop, tooth C}}\approx 30\;{\text{mm}}^{-1}.$$

For teeth A and B, ${\bar{\mu }}_{s,\text{anisotrop}}$ scales approximately linearly with the respective tubule area density. The incisor (tooth C), however, exhibits a lower anisotropic scattering contribution relative to its tubule density.

For comparison, Kienle et al. [8] reported a scattering coefficient of 284 mm^−1^ for light incident at 0° on a single tubule, assuming an areal density of 45 000 mm^−2^. Zijp and Ten Bosch [1] calculated a value of 140 mm^−1^ for a perpendicular array of cylinders with 6 µm spacing. In contrast to these analytical estimates, our values of ${\bar{\mu }}_{\text{anisotrop}}$ represent the effective scattering contribution of dentinal tubules averaged over the full light propagation volume through the dentin tissue.

### 3.2. Evaluation against isotropic-only model

To evaluate the isotropic-only model, we simulated total transmission and reflection for tooth A. In this analysis, only the tooth-slice geometry was considered, omitting the cuvette configuration used in previous measurements. The forward model accounted for anisotropic light propagation based on the tooth’s optical and tubule parameters. Conversely, the inverse model treated the tooth as a homogeneous, plane-parallel slab, with light transport described solely by the absorption coefficient ( $\mu_{a}$ ) and scattering coefficient ( $\mu_{s}$ ). Scattering was modeled using the Henyey-Greenstein phase function with an anisotropy factor of g=0.93 , consistent with reported values for human dentin [17].

**Fig. 4.**
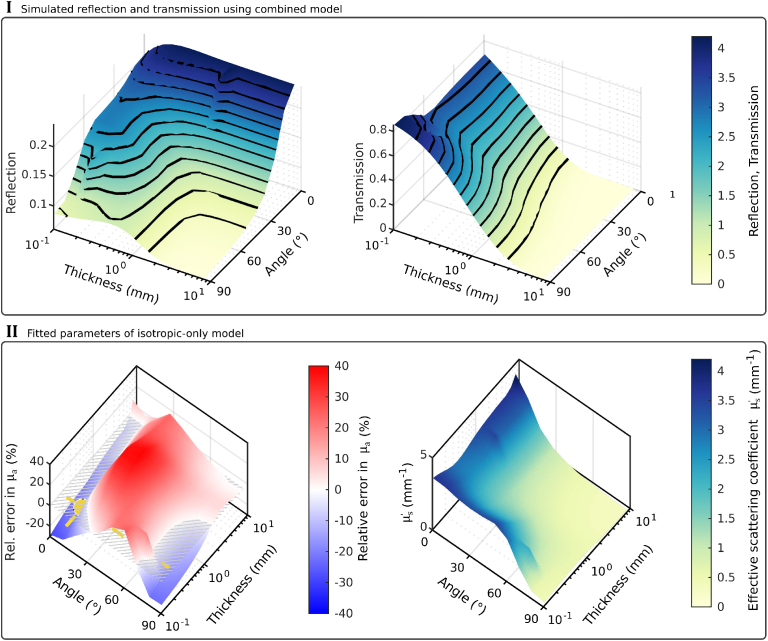
Evaluation of the isotropic-only model **I** Simulated total reflectance and transmittance obtained from the forward model. The isotropic fits (black lines) deviate by less than 1 %. **II** Relative error between the absorption coefficients of isotropic-only fit and those obtained from the anisotropic forward model, together with the fitted effective scattering coefficients.

Figure 4 **I** shows the simulated reflection and transmission for varying tubule thicknesses and tilt angles in the forward model. The deviations between the anisotropic forward simulations and the isotropic fits were less than 1 %, indicating a close match in overall reflectance and transmittance.

Figure 4 **II** presents the absorption and effective scattering coefficients obtained from the isotropic-only fit. The absorption coefficient is shown as the relative error between the isotropic-only fit and the anisotropic forward model. For the original tooth slice (thickness 0.238 mm, forward tilt 12.6°), the relative error of the fitted absorption coefficient was −9.9 %. Across the investigated parameter space, the isotropic-only model exhibited relative errors in the absorption coefficient of up to 40 %, demonstrating that neglecting anisotropic light propagation in dentinal tubules leads to unreliable absorption estimates. The effective scattering coefficient obtained from the isotropic fit for the investigated tooth was $\mu_{s}^{\prime }=2.8\;{\text{mm}}^{-1}$ .

## 4. Conclusion and discussion

We successfully developed and validated a combined model of isotropic and anisotropic light propagation, based on the radiative transfer equation, that quantitatively describes light scattering in human dentin. From goniometric measurements, we extracted optical parameters characteristic of both scattering regimes: the isotropic component arising from inside the intertubular matrix and the anisotropic component from the dentinal tubules. We fitted the intertubular matrix with parameters $\mu_{a}\approx 0.5\;{\text{mm}}^{-1}$ , $\mu_{s}\approx 1.8\;{\text{mm}}^{-1}$ and Henyey Greenstein anisotropy parameter g≈0.85 . Furthermore, we determined geometric parameters describing the tubule microstructure, including their size (approximately 2 µm in diameter) and spatial density (19 000 mm^−2^ to 60 000 mm^−2^). These parameters showed good agreement with both literature values obtained from scanning electron microscopy (SEM) [3,7] and our own observations. Light scattering on dentinal tubules was described by Maxwell’s equations for optically infinitely extended cylinders. In the conducted experiments, tubule orientations were predominantly perpendicular to the incident direction.

A central achievement of this study is that our combined isotropic-anisotropic model not only provides a qualitative description of light propagation in dentin, but also enables robust solution of the inverse problem: uniquely reconstructing key optical parameters and microstructural features from light scattering measurement data. By fitting the model to experimental angular-resolved transmission and reflection curves, we quantitatively determined both the optical properties of the intertubular matrix and the geometric properties of the dentinal tubules. This demonstrates that our approach can extract physically meaningful material parameters directly from optical measurements, thereby validating the model’s physical basis.

Comparison with an isotropic-only model highlights its limitations: absorption coefficients can be misestimated by up to 40 %. The isotropic-only model yields an effective scattering coefficient of $\mu_{s}^{\prime }=2.8\;{\text{mm}}^{-1}$ and scattering coefficient of $\mu_{s}=40\;{\text{mm}}^{-1}$ for tooth A with a fixed anisotropy factor of g=0.93 . These results confirm that accurate analysis of light propagation in human dentin requires accounting for anisotropic scattering by dentinal tubules.

The primary objective of the present study is methodological: to introduce, validate and demonstrate a combined isotropic-anisotropic Monte Carlo framework that allows the inverse problem of dentin optics to be solved uniquely. The three specimens analyzed here are not intended as a comprehensive optical characterization of human dentin, but were deliberately chosen to span a meaningful range of biological and structural variability, including two tooth classes (premolar and incisor), donor ages from 10.4 to 65 years, and tubule areal densities essentially covering the full physiological range reported in the SEM literature [3,5,7]. Future investigations will extend the developed framework to encompass the structural diversity of natural dentition. We plan to characterize optical properties across various tooth classes (incisors, canines, premolars, and molars) and to resolve variations in optical behavior as a function of depth within the dentin (near the enamel–dentin junction, middle dentin, and near the pulp). A systematic mapping of the algorithm’s working envelope across the full parameter space using synthetic forward simulation data is a further important direction for future work. In addition, prior studies have demonstrated that carious lesions produce measurable changes in optical properties [30]. Our model provides a powerful tool for detailed optical characterization of carious lesions, enabling quantitative analysis of how demineralization and structural degradation alter both isotropic and anisotropic scattering properties. Such investigations may contribute to improved early caries detection methods and provide insights into the pathophysiology of dental decay at the optical level.

A natural extension of the present work is the integration of the locally fitted optical and microstructural parameters into whole-tooth simulations. The required geometric framework has recently been demonstrated within our group by Hevisov et al. [31], who implemented a Monte Carlo simulation on a tetrahedral mesh of complete incisor and molar geometries, with the tubule parameters varied per tetrahedron to capture anisotropic light propagation throughout the tooth. In that study, the optical properties of dentin were taken from the literature and treated as approximate reference values. Combining this whole-tooth mesh approach with the experimentally fitted parameters provided by the present framework will enable quantitative, patient-realistic simulations of light transport in entire teeth. Such whole-tooth simulations are a prerequisite for the quantitative interpretation of clinical optical measurements. This is particularly relevant for techniques such as optical coherence tomography, whose contrast in dentin depends on the angle between probe beam and local tubule orientation.

Finally, the model is broadly applicable to biological tissues exhibiting anisotropic light propagation, such as brain white matter, tendon, and muscle tissue, offering a versatile tool for optical characterization beyond dental applications.

## Data Availability

Data underlying the results presented in this paper are available from the corresponding authors upon reasonable request.
